# Intraoperative refractory hypotension in a patient with chronic use of low-dose prednisolone

**DOI:** 10.1186/s40981-019-0307-1

**Published:** 2020-01-03

**Authors:** Shingo Ito, Hiroyuki Seki, Yasushi Innami, Takashi Ouchi

**Affiliations:** Department of Anesthesiology, Tokyo Dental College Ichikawa General Hospital, 5-11-13, Sugano Ichikawa, Chiba, 272-8513 Japan

Chronic steroid therapy may cause hypothalamic-pituitary-adrenal (HPA) axis suppression, posing the potential risk of adrenal insufficiency in the perioperative period. Although there are inconsistent data to accurately predict the degree of adrenal suppression in patients receiving exogenous glucocorticoid therapy, patients treated with low-dose prednisone (≤ 5 mg/day) have been considered to be at low risk of HPA axis suppression [1]. We report a case of intraoperative refractory hypotension in a patient with chronic use of prednisolone 3 mg/day.

A 60-year-old woman (148 cm, 52 kg) underwent open reduction and internal fixation for a femoral neck fracture under general anesthesia. She had been taking prednisolone 3 mg/day for 5 years for the treatment of rheumatoid arthritis. She had a history of hypertension, for which she took calcium-channel blockers, but was otherwise healthy.

On the day of surgery, she took her daily morning dose of prednisolone 3 mg.

Anesthesia was induced with propofol 120 mg and fentanyl 100 μg at 9 a.m. After the laryngeal mask airway was inserted, anesthesia was maintained using desflurane and a mixture of oxygen and nitrous oxide. Her blood pressure decreased from 140/90 to 79/54 mmHg after induction of anesthesia and remained low (systolic blood pressure < 70 mmHg) for 20 min and was unresponsive to repeated administration of ephedrine (a total of 16 mg) and phenylephrine (a total of 0.5 mg). Hydrocortisone 50 mg was administered, owing to the suspicion of adrenal insufficiency. Her systolic blood pressure increased to approximately 90 mmHg within 10 min and was maintained at > 100 mmHg after an additional administration of phenylephrine 0.1 mg. The patient awakened and was discharged from the operating room, soon after the completion of surgery.

In this case, the patient developed refractory hypotension despite taking her daily corticosteroid replacement. Although the adrenocortical function was not assessed, it is probable that the HPA axis was suppressed, as the refractory hypotension rapidly improved after the administration of hydrocortisone. Patients receiving prednisone ≤ 5 mg/day daily have a normal HPA axis response [2]. The recommendations do not recommend the administration of a stress dose of steroid or preoperative testing of adrenal function in these patients unless they exhibit signs of HPA axis suppression [1, 3]. However, a recent study demonstrated that adrenal insufficiency was observed in more than one third of patients during the ongoing use of low-dose prednisolone (5 mg/day) for rheumatoid arthritis [4]. Furthermore, a recent meta-analysis showed that the symptoms of adrenal insufficiency were not observed in most patients with glucocorticoid-induced adrenal insufficiency [5]. While the use of supraphysiologic glucocorticoids may increase the potential risk of steroid-induced side effects such as hyperglycemia, immunosuppression, and impaired wound healing, the results of these studies [4, 5], along with those of the present study, indicate the need for the administration of a stress dose of steroids in some patients with a “low risk” of adrenal insufficiency.

In conclusion, this case showed that under surgical stress, even patients on a low-dose, chronic regimen of prednisolone may require a stress dose of glucocorticoids. The patient provided written informed consent for the publication of this case report.

## Data Availability

Not applicable.

## Abbreviation

FGDs: Focus group discussions

## References

[1] Coursin DB, Wood KE (2002). Corticosteroid supplementation for adrenal insufficiency. JAMA.

[2] LaRochelle GE, LaRochelle AG, Ratner RE, Borenstein DG (1993). Recovery of the hypothalamic-pituitary-adrenal (HPA) axis in patients with rheumatic diseases receiving low-dose prednisone. Am J Med.

[3] Liu MM, Reidy AB, Saatee S, Collard CD (2017). Perioperative steroid management: approaches based on current evidence. Anesthesiology.

[4] Borresen SW, Klose M, Baslund B, Rasmussen AK, Hilsted L, Friis-Hansen L (2017). Adrenal insufficiency is seen in more than one-third of patients during ongoing low-dose prednisolone treatment for rheumatoid arthritis. Eur J Endocrinol.

[5] Broersen LH, Pereira AM, Jorgensen JO, Dekkers OM (2015). Adrenal insufficiency in corticosteroids use: systematic review and meta-analysis. J Clin Endocrinol Metab.

